# Editorial: Endometriosis: a fertility perspective

**DOI:** 10.3389/fendo.2026.1963419

**Published:** 2026-08-25

**Authors:** Irene Iavarone, Carlo Ronsini

**Affiliations:** 1 Department of Woman, Child and General and Specialized Surgery, University of Campania Luigi Vanvitelli, Naples, Italy; 2 Unit of Gynecologic Oncology, National Cancer Institute, IRCCS, Fondazione “G. Pascale”, Naples, Italy

**Keywords:** endometrial receptivity, endometriosis, oocyte quality, ovarian reserve, reproduction

Endometriosis is a chronic, estrogen-dependent inflammatory condition that affects approximately 10% of women of reproductive age; it is a primary driver of female subfertility. The relationship between endometriosis and infertility is multifaceted and involves distorted pelvic anatomy, altered peritoneal function, and endocrine abnormalities that compromise oocyte quality and endometrial receptivity. This Research Topic titled “Endometriosis: A Fertility Perspective” aims to provide a comprehensive overview of the molecular, environmental, and clinical pathways that define reproductive outcomes for patients living with this disease.

Understanding the molecular heterogeneity of endometriosis is essential for advancing precision medicine. Situmorang et al. found that the disease’s three main phenotypes – superficial peritoneal endometriosis (SUP), ovarian endometrioma (OMA), and deep infiltrating endometriosis (DIE) – are driven by distinct gene activation profiles. The authors’ findings suggest that DIE is characterized by invasive fibrotic pathways (e.g., FN1, MMP2), while OMA is dominated by oxidative stress and iron metabolism regulators, reflecting biologically unique entities rather than simple anatomical variations.

Further exploring internal regulatory systems, Olabarrieta et al. investigated the endogenous opioid system as a modulator of uterine physiology. They proposed that dysregulated opioid signaling could impair tissue remodeling, angiogenesis, and apoptosis, closely mirroring the altered mechanisms in endometriosis-associated subfertility. Additionally, the regulation of the corpus luteum (CL) – vital for maintaining early pregnancy – was reviewed by Tariq et al., who detailed how integrated hormonal, metabolic (AMPK–PPARγ), and epigenetic (EZH2-mediated histone methylation) pathways control CL function and potential luteal insufficiency.

The impact of the external environment and systemic health on fertility cannot be overlooked. Iavarone et al. conducted an exploratory study on lead concentrations in follicular fluid and found significantly higher levels in infertile patients with endometriosis compared to controls. This suggests that heavy metal accumulation may parallel the pathogenesis of the disease and negatively affect oocyte quality.

Simultaneously, systemic markers such as Body Mass Index (BMI) have been examined for their predictive value. Park et al. assessed the association between BMI and Anti-Müllerian Hormone (AMH), finding a modest inverse relationship in both endometrioma and dermoid cyst cohorts. The authors’ results underscore that systemic BMI may not fully capture the metabolic influences on ovarian reserve, which are likely influenced more by local ovarian microenvironmental factors in endometriosis.

Ovarian endometriomas pose a direct threat to the reproductive reserve. Orisaka et al. demonstrated through a rat follicle culture model that endometrioma fluid (EmF) disrupts preantral follicle development. Their research identified that EmF induces oxidative stress in granulosa cells and promotes fibrosis in theca cells, thereby impairing the essential cellular crosstalk required for follicular growth.

The management of these cysts remains a clinical dilemma. Li et al. confirmed that endometriotic cystectomy is associated with significantly diminished markers of ovarian reserve (reduced AMH and AFC levels) and poorer responses to controlled ovarian stimulation. These findings highlight the need for individualized surgical decision-making that weighs symptom relief against the risk of iatrogenic damage to ovarian function.

Advancements in technology and pharmacology offer new hope for improving fertility outcomes. Zhang et al. conducted a meta-analysis on the diagnostic accuracy of machine learning models, demonstrating that those based on genetic and imaging information possess substantial precision for the early detection of endometriosis.

In the realm of therapeutics, Sun et al. conducted a systematic review of pharmacological therapies, noting that while hormonal suppression (e.g., dienogest, relugolix) is effective for alleviating pain, the choice of therapy must be tailored to the patient’s specific reproductive goals. For patients seeking non-hormonal, conception-compatible options, Hammond et al. utilized a network meta-analysis to rank VEGF-directed pharmacotherapies. The research team identified compounds such as curcumin that show signals of lesion regression in preclinical models by targeting angiogenesis. This offers a potential route toward fertility-sparing treatment. Complementing these findings, a prospective observational study conducted by Bahar et al. compared dienogest with a levonorgestrel-containing combined oral contraceptive (COC). This study found that while both treatments significantly reduced chronic pelvic pain and dysmenorrhea over 3 months, the COC group experienced greater short-term pain reduction. This suggests that continuous COC regimens may represent a vital, cost-effective conservative treatment option, particularly in resource-limited settings.

For patients with DIE, the sequence of interventions is critical. Aase and Khan reviewed surgical management for fertility enhancement, emphasizing that while surgery can enhance natural fecundity in superficial disease, its role prior to Assisted Reproductive Technology (ART) must be carefully evaluated based on patient age and ovarian reserve.

Optimizing these sequences was the focus of the study by (Tang et al.). The authors reported successful live births in severe DIE cases using a sequential strategy: embryo cryopreservation first, followed by radical laparoscopic surgery, and finally a natural-cycle frozen embryo transfer (NC-FET). This approach prioritizes fertility preservation before extensive surgery, which might otherwise compromise ovarian function. For patients undergoing less invasive procedures, such as ethanol sclerotherapy (EST), Liu et al. developed and validated a clinical nomogram to predict cumulative live birth rates. This tool, based on predictors such as cyst diameter and the number of oocytes retrieved, assists with individualized counseling and shared decision-making.

The research presented in this Research Topic reinforces the view that endometriosis is not a monolithic disease, but rather a complex assembly of biological phenotypes that requires a multidisciplinary, personalized approach. From the molecular impact of endometrioma fluid to the application of machine learning in diagnostics and the strategic sequencing of surgery and ART, these studies advance our understanding of how to manage endometriosis while prioritizing and enhancing a patient’s fertility. These findings provide a robust foundation for future clinical trials and the development of targeted, non-hormonal interventions tailored to the unique needs of women seeking to conceive despite this challenging condition.

